# Contamination Parts and Residue Levels of Multi-Mycotoxins in Medicinal and Edible Locust

**DOI:** 10.3389/fphar.2018.00480

**Published:** 2018-05-17

**Authors:** Dandan Kong, Weijun Kong, Xiaoli Yang, Meihua Yang

**Affiliations:** Key Laboratory of Bioactive Substances and Resources Utilization of Chinese Herbal Medicine, Ministry of Education, Institute of Medicinal Plant Development, Chinese Academy of Medical Sciences – Peking Union Medical College, Beijing, China

**Keywords:** multi-mycotoxins, locust, contamination parts, SEM, HPLC-FLD

## Abstract

Locust is esteemed as a traditional Chinese medicine, as well as one of the most important nutritional foods especially in Asian countries. However, some toxic secondary metabolites such as mycotoxins are usually found in different parts of locust to affect its quality and safety. This study aimed to investigate the aflatoxins (AFs) contaminated parts by observing *Aspergillus flavus*, spores’ diameter, amount and distribution on head, tentacle, wing, belly and shank parts of the locust with scanning electron microscopy (SEM). Furthermore, to assess the residue levels of multi-mycotoxins in the locust, the high performance liquid chromatography with fluorescence detection (HPLC-FLD) was adopted. The technique was used to determine the contents of AFs, zearalenone (ZON) and α-zearalenol (α-ZOL) in locust and the positive samples were confirmed by high performance liquid chromatography-tandem mass spectrometry (HPLC-MS/MS). The chromatographic conditions, MS/MS parameters and the method of sample extraction were carefully optimized. Results revealed that obvious differences of *Aspergillus flavus* strains and spores were found, while the spores’ diameter ranged from 3.0 to 13.0 μm in different contaminated parts of the locust samples. The HPLC-FLD method for multi-mycotoxins analysis showed good selectivity, linearity, recovery and precision. Limits of quantification (LOQs) were lower than 27.6 μg/kg, while limits of detection (LODs) were in the range of 0.02–8.6 μg/kg. The accuracy of the developed method was validated regarding recoveries of 80.1–118.1% with relative standard deviation (RSD) ≤ 11.4%. Finally, the developed multi-mycotoxin method was applied for screening of these mycotoxins in 11 commercial locust samples. Only AFB_1_ and AFB_2_ were found in six samples, and the contamination levels ranged from 0.12 to 4.4 μg/kg, which were lower than the maximum residue limit and can be used safely. This is the first report on the exploration of contamination parts and levels of multi-mycotoxins in medicinal and edible locust. The combined method of SEM and HPLC-FLD exhibited advantages of low cost, high sensitivity, rapid determination, convenience and especially intuitive judgment, which is proposed for contamination parts observation, for the large-scale quantification of multi-mycotoxins in other medicinal animal matrices.

## Introduction

Locust species mainly include *Locusta migratoria manilensis* (Meyen) and *Oxya chinensis*, which contain a high nutritional composition, such as protein, lipid, trace elements, and vitamins (Hinks et al., 1993; Wang et al., 2007). As a traditional Chinese medicine (TCM), according to the reported literatures, locust is beneficial for the treatment of cough, asthma, infantile convulsion, detumescence, pain regarding the effect of strengthening tonics for thousands of years (Buszewska-Forajta et al., 2015). Locust is also expected to be a new candidate of antibiotics, and antitumor drugs because of the abundant hormone, antifreeze proteins, and storage proteins (Fürst, 2016). However, contaminants such as toxic mycotoxins in locust have greatly affected its quality and safety, as well as the health of consumers, which has become a severe problem (Zhang et al., 2010).

A great amount of animals are reported to be easily contaminated by zearalenone (ZON) and its metabolite of α-zearalenol (α-ZOL) after biting the leaves and stems of gramineous plants that are polluted by ZON, such as wheat, maize, oats, sorghum, rice, millet, and reeds (D’Mello et al., 1999; Döll and Dänicke, 2011). Aflatoxins (AFs) should be strictly required to be detected in TCMs, including animal-origin TCMs, especially under high temperature and humidity conditions due to unbefitting transportation or storage or when stored with contaminated medicinal materials (Shehu and Bello, 2011; Chinese Pharmacopoeia Commission, 2015). Among these mycotoxins, aflatoxin B_1_ (AFB_1_) was recorded in 1961 for the first time with the most potent of carcinogenicity to cause animal death extensively, which has been classified as group I by the International Agency for Research on Cancer (IARC) (IARC, 1993). Epidemiological studies have shown a good correlation between AFB_1_ intake and cancer of some organs (Groopman et al., 1988). ZON and α-ZOL are stable during storage, milling, processing, and cooking, which is thermo-stable and possesses estrogenic properties (EFSA, 2004) with strong effects on immune system, protein synthesis, steroid synthesis, embryonic development and with the alteration of liver, spleen, and uterus (Döll and Dänicke, 2011; Escrivá et al., 2015). However, the three mycotoxins are also widely and naturally distributed in agriculture commodities and foods, which may significantly threaten the health of human beings (El-Barbary, 2015; Zhao et al., 2018). Due to the carcinogenicity, teratogenicity, mutagenicity, and immunosuppressive activity of AFs, ZON, and α-ZOL, some countries and organizations have set the maximum limits in foodstuffs, feeds, and other matrices intended for human or animal consumption (European Commission, 2010). It is worth mentioning that the maximum residue limit of AFs has been restricted to 5 μg/kg in the Chinese Pharmacopoeia (Chinese Pharmacopoeia Commission, 2015), and 75–400 μg/kg in cereals, bakery products and maize oil set by European Union (European Commission, 2010).

Thus, it is important to find out a desirable method for routinely detecting the contamination levels of multi-mycotoxins in foods, TCMs and other matrices before their consumption. Traditional methods for determining mycotoxins have been divided into two categories: reference methods for quantitative analysis and rapid methods for fast screening of large-scale samples. High performance liquid chromatography-tandem mass spectrometry (HPLC-MS/MS) with indisputable advantages like high sensitivity, selectivity, accuracy, and throughput has been the preferred method for multi-residue analysis, but is limited to its high price and complicated operation (Streit et al., 2013; Wang et al., 2013). Besides, high performance liquid chromatography coupled with fluorescence detection (HPLC-FLD) has achieved the large range determination of multi-mycotoxins with the higher repeatability and lower cost than HPLC-MS/MS (Golge et al., 2016; Cao et al., 2017). However, up to now, there’s no research concerning the analysis of multi-mycotoxins in the matrices of medicinal animals such as locust published.

Current studies of accumulated mycotoxins are mainly focused on detection method (Girolamo et al., 2017; Myndrul et al., 2017), toxicology (Devi et al., 2017), decontamination (Patriarca and Pinto, 2017), quality influence (Piacentini et al., 2017), precaution in storage process and risk assessment (Jacxsens et al., 2016; Oyedele et al., 2017). Further studies should be carried out in the separated tissue and its molecules for investigating the curative components (Liang et al., 2013), fungus growth and mycotoxins production in medicinal plants by laser micro dissection which are widely used in the size of cellular membranes with the characteristics as time-consuming, expensive and tedious (Chandran et al., 2011). Besides, few studies have been reported on the contamination parts in microstructure size on different parts of medicinal animals with much protein and fat that might be necessary for the growth of *Aspergillus flavus* (Casquete et al., 2017; Liu et al., 2017). Moreover, the research on the contamination parts of *Aspergillus flavus* growth in medicinal animals have directive significance to the prevention and control of large-scale AFs pollution, the research on distinction in different matrices and suggestion of medicinal parts.

Therefore, the aim of this study is to profile in depth the contamination parts and residue levels of AFs in locust and to establish a desirable method to simultaneously analyze multi-mycotoxins in this medicinal animal matrix by using the combined method of SEM and HPLC-FLD after post-column derivatization. To the best of our knowledge, this is the first report for intuitive judgment of contamination parts and especially simultaneous analysis of multi-mycotoxins in locust, providing significant reference and guidance for economical and convenient analysis of multi-mycotoxins in other animal matrices.

## Materials and Methods

### Animal Welfare

This study was conducted in compliance with the Declaration of Helsinki, and the research protocol was followed closely to Guide for the Care and Use of Laboratory Animals although there’s no provision to show that invertebrates need to be treated by animal welfare. All live locusts reared under a 14:10 light/dark photo regime at 30 ± 2°C and fed on a diet of fresh greenhouse-grown wheat seedlings and wheat bran. Live locust samples used for cultivating *Aspergillus flavus* were newly executed after anesthesia, and all efforts were made to minimize suffering.

### Chemicals, Products, and Reagents

The standard spore suspension of *Aspergillus flavus* was purchased from China General Microbiological Culture Collection Center (Beijing, China). The analytical standards of ZON and α-ZOL toxins in powder form (5 mg) were purchased from Sigma Aldrich (St. Louis, MO, United States) and mixed standard solution of AFB_1_ and AFG_1_ (each 1.0 mg/mL), AFB_2_ and AFG_2_ (each 0.3 mg/mL) were all supplied by SUPELCO (Bellefonte, PA, United States). Methanol used as the mobile phase and extraction solvent were of HPLC grade and provided by Honeywell (Morristown, NJ, United States). Sodium chloride, disodium hydrogen phosphate, potassium chloride, potassium dihydrogen phosphate were all guaranteed grade and obtained from Chinese Medicine Group Chemical Reagent Co., Ltd. (Beijing, China). A KQ-500 ultrasonic-assisted extraction apparatus was supplied by Kunshan Ultrasonic Instrument Co., Ltd. (China). Immunoaffinity columns were attached with both AFs and ZON and supplied by NuClover (China). GF/A glass microfiber filters (1.5 μm) were obtained from Vicam (Watertown, MA, United States). Purified water (Wahaha, Hangzhou, China) was used throughout the analysis. The live locust used in the experiment was collected from Guangxi province, China. Locust medicinal samples were purchased from pharmacies in China in September, 2011 and were homogenized by using laboratory mill, then passed through filter (F¼ 250 ± 9.9 μm, IKA, Germany) and stored in valve bags at -20°C before use.

### Preparation of Standard and Other Solutions

The fungal spore suspension was prepared according to the reported method (Yang et al., 2017). And the concentration of spore suspension was adjusted to 10^7^ cfu/mL with a hemocytometer slide (0.1 mm depth, 1/400 mm^2^) and an XDS-1B optical microscope (Chongqing, China) to get the standard spore suspension for subsequent use (Li et al., 2016). Stock solutions of ZON and α-ZOL were prepared in methanol (50 μg/mL) and stored at 20°C. A mixed working standard solution including AFB_1_, AFB_2_, AFG_1_, AFG_2_, ZON, and α-ZOL was prepared by combining suitable aliquots of each individual stock solution, followed by diluting with appropriate amounts of methanol. Then, the standard solutions were protected from light and stored in screw capped glass tubes, which were wrapped with parafilm and stored at -20°C. Phosphate buffer (pH 7.0) was prepared by mixing sodium chloride (8 g), disodium hydrogen phosphate (2.9 g), potassium chloride (0.2 g), and potassium dihydrogen phosphate (0.24 g) and then diluted with pure water to 1 L.

### Sample Extraction and Clean-Up

A portion of 5.0 g homogenized sample was blended with 25 mL of acetonitrile–water (84: 16, ν/ν) and 1 g NaCl. The mixture was shaken for 5 min by vortex and extracted in an ultrasonic apparatus for 15 min, then filtered using a pre-folded filter paper. 10 mL of the extracted filtrate was diluted with 40 mL of PBS solution, and shaken vigorously. Then, the blended filtrate was passed through the glass microfible filter. Next, 25 mL of the filtrate was passed through an immunoaffinity column (IAC, attached both AFs and ZON) and the target mycotoxins were absorbed on the column. The column was washed three times with 20 mL of PBS solution (pH 7.0) until 2–3 mL of air entirely passed through the column. The mycotoxins bound to the specific antibody needed to be eluted by passing 3 mL of methanol through the column and collected in a HPLC vial, and then the eluent should be concentrated to 0.5 mL by nitrogen at 45°C. Finally, the eluate should be diluted with 0.5 mL of ultrapure water and maintained at 4–8°C in the HPLC vial before use (Liu et al., 2016).

### Instrumentation

The analysis of contamination level of multi-mycotoxins in locust was carried out on a HPLC (LC-20AT, Shimadzu, Kyoto, Japan) system, which connected with a fluorescence detector (FLD) (RF-10AXL, Shimadzu, Kyoto, Japan) and a photochemical derivative instrument (Aura Industries, Shimadzu, Kyoto, Japan). The chromatographic separations of AFs, ZON and α-ZOL were performed on a ProntoSIL Kroma Plus C18 column (250 mm × 4.6 mm × 5 μm, Bischoff, German). Post-column derivatization was performed through a photochemical derivative apparatus to enhance the fluorescence intensity of all mycotoxins. Fifty microliters of sample solution was injected into the column, while the oven temperature was set at 25°C and the flow rate was 1 mL/min. Methanol (A)-water (B) system was used as the gradient elution solution and 50–68% A was adopted before 12 min, then 68% A was adopted from 12 to 25 min. The FLD was set to the excitation and emission wavelengths of 360 nm and 440 nm before 12 min, and of 280 nm and 440 nm from 12 to 25 min respectively.

LC-MS/MS (Agilent LC 1290-6490, triple quadrupole) analysis was used for the confirmation of mycotoxins in positive sample and the relevant parameters were shown in **Tables 1**, **2**. The analysis of four AFs was operated by the HPLC system on a Poroshell 120 EC-C_18_ (2.1 × 100 mm, 2.7 μm) column and the relevant parameters were also shown in **Tables 1**, **2**. 10 μL of sample solution was injected into the column, while mobile phase solution A was water (0.1% formic acid and 5 mM ammonium acetate) and B was methanol.

**Table 1:** Parameters of flow rate and the conditions of mobile phase gradient for detection of four AFs by HPLC-MS/MS.

Time/min	Flow rate/(mL/min)	A (%)	B (%)
0.00	0.4	90	10
0.50	0.4	90	10
3.00	0.4	60	40
6.00	0.4	55	45
7.00	0.4	10	90
8.00	0.4	10	90
8.10	0.4	90	10


**Table 2:** Instrument parameters of MS/MS conditions for detection of four AFs.

Components	AFG_2_	AFG_1_	AFB_2_	AFB_1_
Precursor ions Q1 (m/z)	331.2	329.2	315.2	313.2
Product ions Q3 (m/z)	313.1	311.1	287.1	285.1
	245.1	243.1	259.1	241.1
Capillary voltage (V)	3,500	3,500	3,500	3,500
Fragmentor (V)	380	380	380	380
Collision energy (V)	25	20	25	22
Nebulizer temperature (°C)	150	150	150	150
Nebulizer pressure (psi)	45	45	45	45
Nebulizer flow rate (L/min)	15	15	15	15
Sheath temperature (°C)	350	350	350	350
Sheath flow rate (L/min)	11	11	11	11


### Method Validation

The analytical method was validated in terms of linearity, limit of detection (LOD), limit of quantification (LOQ), recovery, repeatability, precision, stability, and uncertainty for specific determination of multi-mycotoxins (Kong et al., 2013). To assess linearity, calibration curves were formed by calculating the peak area of analytes at three different concentrations ranging from 0.06 to 6.0 ng/mL for AFG_2_ and AFB_2_, from 0.2 to 20.0 ng/mL for AFG_1_ and AFB_1_, and from 30.0 to 600.0 ng/mL for α-ZOL and ZON *versus* the corresponding concentration in the mobile phase. The linearity was determined by linear regression analysis and coefficient of determination (*R*^2^) value of >0.99 for each mycotoxin was acceptable. The LOD and LOQ were calculated based on the equation of S/N ≥ 3 and ≥10 respectively. For intra-day variation, the locust extract was analyzed in a single day under the same conditions with short time interval, whereas for inter-day variation, the extract was detected once within six consecutive days by comparing recoveries. Recoveries and repeatability were tested by using non-contaminated (mycotoxins-free) locust samples spiked with 1.0, 5.0, and 10.0 μg/kg of AFB_1_ and AFG_1_ while 0.3, 1.5, and 3.0 μg/kg of AFB_2_ and AFG_2_, and 30.0, 60.0, and 300.0 μg/kg of α-ZOL and ZON. All the results were measured in three independent replicates.

### Sample Inoculation, Morphological Observation, and Detection of Multi-Mycotoxins in Locust Samples

Locust sample was divided into five parts and sterilized under a UV lamp overnight. Then the locust were placed in five culture dishes and 1 mL of homogeneous standard spore suspension of *Aspergillus flavus* and 10 mL sterile water were added into the center of the dishes, and all dishes were carefully sealed by parafilm and incubated for 7 days under the temperature of 30°C and humidity of 80%. Then, each part of locust was subjected to critical-point drying by CO_2_ and sprayed with gold in a metallizer and was respectively analyzed for the further morphological and microcosmic observation by using a scanning electron microscope (SEM, Zeiss SUPRA 55 with an accelerating voltage of 20 kV). *Aspergillus flavus* and its spores were observed with different magnifications from 500 to 10,000. The optimized analytical method was applied to detect the contamination level of multi-mycotoxins in the whole locust samples. In total, 11 samples from Yunnan, Guangxi, and Anhui provinces, China were compared.

## Results and Discussion

### Contamination Parts of Locust by Aspergillus Flavus Through Morphology Observation

**FIGURE 1:**
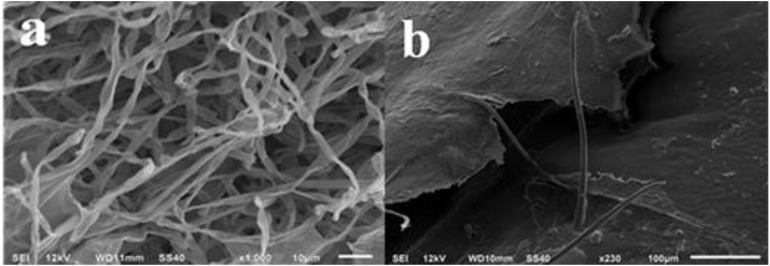
SEM images of **(a)** head, and **(b)** belly surface of spore strains on locust after cultivation with *Aspergillus flavus* for 7 days.

**FIGURE 2:**
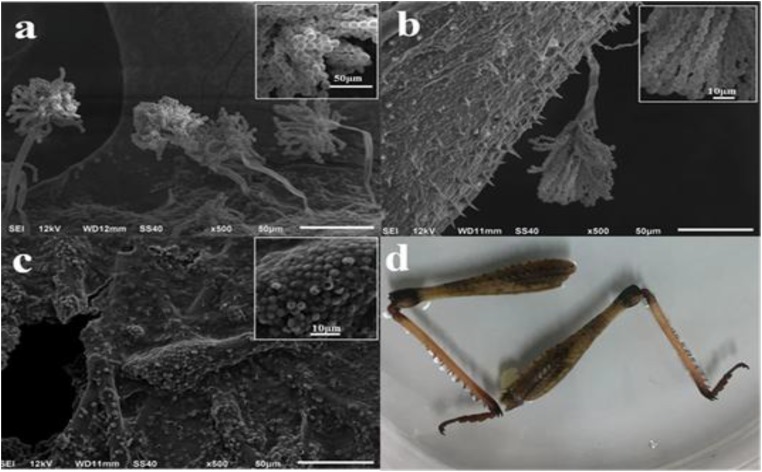
SEM images of spores on locust and facade image after cultivation with *Aspergillus flavus* for 7 days. **(a)** Tentacle; **(b)** wing; **(c,d)** shank.

After adding spore suspension onto different parts of locust for a 7-day cultivation under the optimized conditions, plentiful aflatoxigenic fungi such as *Aspergillus flavus* were widely spread over the parts of head, tentacle, wing, belly and shank of locust. From the SEM micrograph images, no particularly apparent distinctions were found in the growth of aflatoxigenic fungi with different parts of locust. However, it is clearly observable that the hypha was overlapped intricately in the parts of head and belly surface (**Figure 1**). As shown in **Figure 2**, the spores and strains of *Aspergillus flavus* were clearly adhered to the surface of the tentacle, wing and shank, while the amount of *Aspergillus flavus* was especially large in the tentacle part sample. The spores in the wing and shank samples had lower amounts and smaller diameters (4.0–8.0 μm and 3.0–5.0 μm, respectively) compared to the spores of the tentacle sample (10.0–13.0 μm). Thus, the conclusion could be reached that the matrix of the tentacle is suitable for growing the aflatoxigenic fungi, even though there is less fat and protein on the wing and tentacle than the head, belly and shank.

Although the spores and strains of *Aspergillus flavus* were clearly visible and existed in large amounts on different parts of the locust by SEM, it was difficult to judge accurately whether the locust was contaminated with AFs on superficial characteristics as were shown in **Figure 2d**. According to the production mechanisms of mycotoxins and the morphology data above, if only one part of locust was contaminated, the medicine needs to be abandoned or detected for the contamination level strictly before consumption. (Adebajo et al., 1994; Casquete et al., 2017; Devi et al., 2017).

### Method Validation

#### Selectivity

**FIGURE 3:**
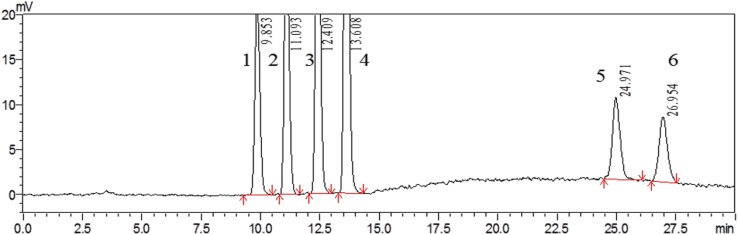
HPLC chromatogram of non-contaminated locust extract after being spiked with mixed standard solution containing 3 μg/kg of AFG_2_ (peak 1), 10 μg/kg of AFG_1_ (peak 2), 3 μg/kg of AFB_2_ (peak 3), 10 μg/kg of AFB_1_ (peak 4), 300 μg/kg of α-ZOL (peak 5), and 300 μg/kg of ZON (peak 6).

The IACs were used for better cleaning-up of the extraction solution, a highly selective FLD, post-column derivatization facilitates and the correct identification of the analytes all led to a further improvement of selectivity of the method. Based on the above conditions, the selectivity of the method was evaluated by analyzing the extract of non-contaminated (mycotoxins-free) locust and a mixture of tested mycotoxins standards under the above-optimized HPLC-FLD conditions. No interfering peaks were observed at the retention time of each analyte when the blank samples were analyzed. The retention time was 9.85, 11.09, 12.41, 13.61, 24.97, and 26.95 min for AFG_2_, AFG_1_, AFB_2_, AFB_1_, α-ZOL, and ZON, respectively (**Figure 3**). The above results demonstrated good selectivity of the developed HPLC-FLD method.

#### Linearity, LOQ, and LOD

Different volumes of working standard solutions were added to the blank extract of locust and analyzed in triplicate. The calibration curve was constructed by plotting the peak area ratio of each analyte *versus* each concentration. Good linear relationships and satisfactory coefficients of determination were achieved for the calibration curves of multi-mycotoxins (**Table 3**). Calibration curves fitted by linear regression showed coefficients of determination (*R*^2^) ranging from 0.9997 to 0.999. And, the LODs were 0.04 μg/kg for AFG_2_ and AFB_1_, 0.10 μg/kg for AFG_1_, 0.02 μg/kg for AFB_2_, 6.60 μg/kg for α-ZOL, and 8.60 μg/kg for ZON. While LOQs were 0.14 μg/kg for AFG_2_ and AFB_1_, 0.32 μg/kg for AFG_1_, 0.07 μg/kg for AFB_2_, 19.10 μg/kg for α-ZOL, and 27.60 μg/kg for ZON. Therefore, the analytical performance indicated that the proposed method can be applied in the determination of trace multiple mycotoxins in locust.

**Table 3:** Validation data of AFG_2_, AFG_1_, AFB_2_, AFB_1_, α-ZOL, ZON.

Mycotoxin	Liner range (ng/mL)	Equation	*R*^2^	LOD (μg/kg)	LOQ (μg/kg)
AFG_2_	0.06–6.00	*y* = 87759*x* + 957.6	0.9999	0.04	0.14
AFG_1_	0.20–20.00	*y* = 36771*x* – 1577.6	0.9999	0.10	0.32
AFB_2_	0.06–6.00	*y* = 152531*x* + 3367.8	0.9998	0.02	0.07
AFB_1_	0.20–20.00	*y* = 74287*x* + 6625.6	0.9998	0.04	0.14
α-ZOL	30.00–600.00	*y* = 688.2*x* + 5560.3	0.9999	6.60	19.10
ZON	30.00–600.00	*y* = 679.8*x* + 703.1	0.9997	8.60	27.60


#### Precision, Stability, and Accuracy

To examine whether the data provided by HPLC-FLD was precise, intra- and inter-day precision data of mycotoxins were shown in **Table 4**, and it can be observed that the RSDs ranged from 0.60 to 1.21% for intra-day and 0.57 to 3.90% for inter-day, proving a good precision. The stability was evaluated by injecting and analyzing the analytes at 0, 2, 4, 6, 8, and 12 h under the same condition in 1 day. RSD values of integral peak areas were also shown in **Table 4**, and the data ranged from 1.03 to 1.82%, which further proved that the optimized method was satisfactory regarding stability within 12 h. For accuracy assessment, the method was assessed through recovery and corresponding RSD, and the results obtained by spiked with the mixed standard solution into non-contaminated locust extract in triplicate at three different levels (low, intermediate, and high). As was shown in **Table 5**, the recoveries were between 80.1 and 118.1%, and RSD values were acceptable as they were below 11.4% for all mycotoxins which were in good agreement with the performance criteria requirement of the EC (European Commission, 2010).

**Table 4:** Intra- and inter-day precision of the developed method.

Concentration of reference solution (ng/mL)	Intra-day RSD (%)	Inter-day RSD (%)	Stability RSD (%)
AFG_2_ (0.6 ng/mL)	0.80	0.62	1.03
AFG_1_ (2.0 ng/mL)	0.99	0.98	1.53
AFB_2_ (0.6 ng/mL)	0.60	0.57	1.11
AFB_1_ (2.0 ng/mL)	0.96	0.87	1.82
α-ZOL (60.0 ng/mL)	1.19	3.90	1.56
ZON (60.0 ng/mL)	1.21	3.12	1.64


**Table 5:** Recovery and repeatability of AFG_2_, AFG_1_, AFB_2_, AFB_1_, α-ZOL, ZON.

Mycotoxins	Spiked level (μg/kg)	Test value (μg/kg)			Recovery/(%, *n* = 3)	RSD (%)
				
		1	2	3
AFG_2_	0.3	0.32	0.34	0.33	110.6	7.50
	1.5	1.36	1.37	1.34	90.3	1.15
	3	2.40	2.55	2.53	83.1	3.10
AFG_1_	1	1.06	1.08	1.03	105.7	8.33
	5	4.37	4.49	4.37	88.3	1.27
	10	8.49	8.94	8.82	87.6	2.67
AFB_2_	0.3	0.30	0.32	0.30	102.8	9.61
	1.5	1.33	1.38	1.31	89.4	2.65
	3	2.61	2.74	2.68	89.2	2.41
AFB_1_	1	1.06	0.93	0.95	97.7	11.4
	5	4.52	4.64	4.41	90.5	2.55
	10	8.90	9.53	9.41	93.1	3.01
α-ZOL	30	35.73	34.91	35.62	118.1	7.87
	60	55.62	50.66	57.73	91.1	6.64
	300	298.67	288.82	266.88	94.9	5.71
ZON	30	32.47	34.27	29.01	106.4	8.38
	60	49.73	47.81	46.62	80.1	3.27
	300	293.59	344.65	313.89	105.8	8.10


### Detection of Multi-Mycotoxins in Locust Samples and Confirmation by LC-MS/MS

**FIGURE 4:**
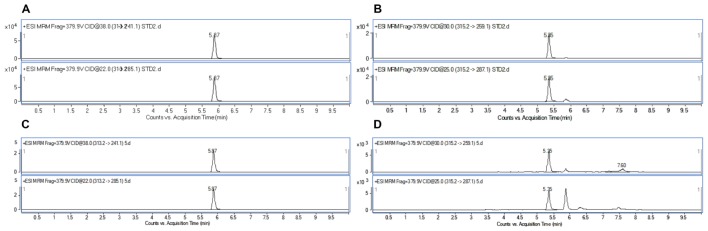
HPLC/MRM-chromatograms for synchronous confirmation of AFB_1_ and AFB_2_ in locust sample. **(A)** AFB_1_ standard solution; **(B)** AFB_2_ standard solution; **(C)** No. 1 sample contaminated with AFB_1_; **(D)** No. 8 sample contaminated with AFB_2_.

**FIGURE 5:**
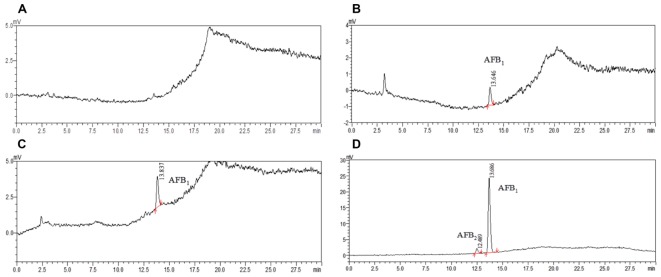
The HPLC chromatograms of commercially available samples. **(A)** Non-contaminated locust (NO. 7 and No. 3); **(B)** locust sample (No. 1 from Guangxi province); **(C)** locust sample (No. 9 from Bozhou city); **(D)** locust sample (No. 8 from Bozhou city).

To demonstrate the applicability of the developed HPLC-FLD method, 11 batches of commercial locust samples including three main production areas in China were analyzed for the target multi-mycotoxins. Moreover, due to the complex matrix of locust and for avoiding the false-positive result, the positive No. 8 and No. 1 samples were selected for further confirmation by LC-MS/MS. As shown in **Figure 4**, the precursor ions and its product ions of AFB_1_ and AFB_2_ in contaminated samples were in good accordance with that of the corresponding standard solutions. The results showed that residual levels of mycotoxins in most of the locust samples were below LODs or LOQs. As were shown in **Table 6** and **Figure 5**, no AFG_1_, AFG_2_, α-ZOL, or ZON was detected in all samples, but AFB_1_ was present in Nos. 1, 5, 8, 9, 10, and 11 samples with the concentration level ranging from 0.14 to 4.4 μg/kg and AFB_2_ was only present in No. 8 sample with the concentration of 0.12 μg/kg. However, except for No. 8 sample from Anhui Province, the contamination levels of mycotoxins were all lower than the regulatory MRLs suggested by EU (European Pharmacopeia Committee, 2013) and China (Chinese Pharmacopoeia Commission, 2015). All the detected mycotoxins and positive samples were confirmed by LC-MS/MS analysis.

**Table 6:** Co-detection of six mycotoxins in real locust samples from different sources in China (*n* = 3).

No.	Source	Concentration (μg/kg)					
		
		AFG_2_	AFG_1_	AFB_2_	AFB_1_	α-ZOL	ZON
1	Guangxi	– ^a^	–	–	0.17	–	–
2	Guangxi	–	–	–	<LOQ ^b^	–	–
3	Guangxi	–	–	–	–	–	–
4	Guangxi	–	–	–	<LOQ	–	–
5	Yunnan	–	–	–	0.14	–	–
6	Yunnan	–	–	–	<LOQ	–	–
7	Yunnan	–	–	–	–	–	–
8	Bozhou	–	–	0.12	4.4	–	–
9	Bozhou	–	–	–	0.32	–	–
10	Bozhou	–	–	–	0.46	–	–
11	Bozhou	–	–	–	0.69	–	–


*^a^Concentration cannot be detected in locust samples. ^b^Detected concentration lower than LOQ.*

In consideration of such serious toxicity and high incidence rate of these mycotoxins, it is of great importance to establish a reliable method for detecting mycotoxins in locust. Through the investigation of the contamination parts of locust, one could found that if a part of locust was detected with mycotoxins, the fungi would sustainably propagate resulting in the accumulation of mycotoxins in the following processes of transportation or storage. For guaranteeing the quality and safety, locust needs to be checked carefully before use or is prohibited to use directly.

## Conclusion

A sensitive and selective HPLC-FLD method was developed and validated for the co-detection of multi-mycotoxins in locust, which showed good selectivity, linearity, recovery, and precision. In order to clarify the contamination parts of AFs in locust, the *Aspergillus flavus* was incubated and observed in every specific part of locust. The morphologies of strains and spore’s diameter changes were carefully investigated by SEM and the results revealed that there’s no obvious distinctions in different parts such as wing, head, belly, tentacle, and shank. However, it can be easily concluded that if one part of the locust was contaminated with AFs, the samples needed to be prohibited for usage. Furthermore, it’s meaningful for aiding the exploration of special contaminated parts in other matrix of animal medicines. Besides, the proposed method has been successfully applied to the co-detection of these mycotoxins in 11 commercial locust samples. Only AFs was found in six samples with the contamination levels ranging from 0.12 to 4.40 μg/kg. With the advantages of high sensitivity, rapid determination, convenience and especially avoiding the intuitive judgment, the combined method of SEM and HPLC-FLD will definitely make contributions to the progress toward the rapid screening of large-scale detection and quantification of multiple mycotoxins in other medicinal animal matrices.

## Author Contributions

DK and XY performed the research. MY designed and performed the experiments and the research study. DK and WK analyzed the data. DK wrote the paper.

## Conflict of Interest Statement

The authors declare that the research was conducted in the absence of any commercial or financial relationships that could be construed as a potential conflict of interest.
